# Simon Fraser University Speech Error Database (SFUSED) Cantonese: Methods, design, and usage

**DOI:** 10.3389/fpsyg.2024.1270433

**Published:** 2024-01-25

**Authors:** John Alderete

**Affiliations:** Linguistics and Cognitive Science, Simon Fraser University, Burnaby, BC, Canada

**Keywords:** speech errors, Cantonese, data collection, data quality, language production, ecological validity

## Abstract

The Simon Fraser University Speech Error Database (SFUSED) is a multi-purpose database of speech errors based in audio recordings. The motivation for SFUSED Cantonese, a component of this database, is to create a linguistically rich data set for exploring language production processes in Cantonese, an under-studied language. We describe in detail the methods used to collect, analyze, and explore the database, including details of team workflows, time budgets, data quality, and explicit linguistic and processing assumptions. In addition to showing how to use the database, this account supports future research with a template for investigating additional under-studied languages, and it gives fresh perspective on the benefits and drawbacks of collecting speech error data from spontaneous speech. All of the data and supporting materials are available as open access data sets.

## 1 Introduction

This report documents the methods and some of the results from SFUSED Cantonese, a database of 2,502 speech errors collected from conversations in Cantonese (accessible from: https://osf.io/u58m9/). The speech errors were collected from audio recordings and cross-classified with over 70 variables (fields) designed to support linguistic and psycholinguistic research. As a relatively large database of a language with no prior speech error study, SFUSED Cantonese can help provide a broader cross-linguistic picture of language production, a research area that has historically been skewed toward Indo-European languages (Berg, 1987; Chen et al., 2002; Costa et al., 2007; Griffin and Crew, 2012; Alderete and O’Séaghdha, 2022). SFUSED Cantonese has already contributed to our understanding of syllable and tone encoding in Chinese languages (Alderete et al., 2019), the linguistic analysis of Cantonese tone (Alderete et al., 2022), and sub-lexical encoding in Cantonese (Alderete, 2022). We hope that this account of its methods can support new research endeavors.

Despite this potential, there is a sense that our contribution is swimming against the tide of experimental research in language production. Since the 1980s, the scientific study of speech errors, and psycholinguistics in general, has shifted away from research in public settings and moved into the laboratory (Baars, 1992; Stemberger, 1992; Bock and Huitema, 2016). While most recognize the important early contributions of speech error research (Fromkin, 1971; Garrett, 1975; Shattuck-Hufnagel, 1979; Stemberger, 1982/1985), the results of corpus studies are sometimes viewed with skepticism. Speech errors collected from spontaneous speech may have skewed distributions because of perceptual biases (Ferber, 1991; Pouplier and Goldstein, 2005; Pérez et al., 2007), they have phonetic structure and variability that is difficult to document (Frisch and Wright, 2002; Katsika et al., 2014), they exhibit many problems with classification and analysis (Cutler, 1988), and they can have poor data quality because of missed or misheard errors (Cutler, 1982; Ferber, 1995). In addition, speech errors are tremendously time-consuming to collect and analyze (Bock, 1996), and so large collections that require many years to produce can be difficult to justify. In this context, one might reasonably ask, why are we still collecting speech errors from natural speech?

In 2006, the Linguistic Society of America organized an institute workshop to discuss the state of the art in speech error research and address many of these issues (Schütze and Ferreira, 2007a). The summary of this meeting (Schütze and Ferreira, 2007b) re-affirmed the conclusion that naturalistic speech errors provide crucial insights into language production processes, and these insights are unique because they provide certain kinds of evidence that cannot be produced in the lab. This positive conclusion has been reinforced since this meeting by a number of studies, including new technology for crowd-sourcing speech error collection (Vitevitch et al., 2015), several new speech error studies of non-Indo-European languages (Wan, 2007; Liu, 2009; Han et al., 2019; El-Zawawy, 2021), and contemporary discussion of the value of spontaneous speech error data (Terao, 2022). However, despite this enthusiasm, the meeting also brought two important problems with corpus studies into sharper focus, namely the non-random distribution of errors and the time commitment that collecting speech errors in the wild requires to produce solid results.

In many ways, the construction of SFUSED Cantonese, and its cousin database SFUSED English (Alderete, 2019), delivers directly on the concerns of this meeting. On data quality, the techniques we have developed and tested significantly boost sample coverage and reduce skewing in speech error patterns (Alderete and Davies, 2019), and we demonstrate this below for the first time in SFUSED Cantonese. In addition, we show that, through the existence of explicit management systems, our methods significantly reduce the amount of time to produce a large-scale database. With rather generous estimates of our time commitment, the creation of SFUSED Cantonese requires approximately the same amount of time that a small lab would otherwise spend on developing and deploying any new research tool. When one considers the richness of the linguistic data, the access to the underlying speech, and the potential for multi-purpose use of the database, we feel the case for continuing speech error collection is incredibly strong. Because of these benefits, and the lack of detailed accounts of the methods for collecting and classifying speech errors in general [but see Stemberger (1982/1985) and Jaeger (2005)], we dedicate some space to explaining the specific procedures that we used so that future work can build on it.

The rest of this article is organized as follows. The next section fleshes out the detailed methods used to build the database, including linguistic research, training, data collection, and classification, and it ends with an overall assessment of the data quality of the speech errors in the database. Section 3 goes on to describe the structure and logic of longform entries in the database, showing users how to interpret the markup and some of the fields in the database. Sections 4 explains the linguistic assumptions we used to make the database, including describing the sound, word, and phrase structures of Cantonese. Section 5 explains our processing assumptions, addressing subtle but important issues concerning classification, error ambiguity, the context and lexicality of speech errors, and the semantics of relationships between intended and error words. In section 6, we illustrate two basic ways to use the database, the longform interface for drilling down into individual speech error records and the dimensions interface to investigate research questions from a global view of the database. Section 7 concludes with some sketches of how the structure of the database might be used to support future research.

## 2 Methods

### 2.1 Overview of the stages

The stages described in Table 1 can be thought of as a set of sub-systems in a larger workflow, where each stage is designed to produce a particular outcome for the next stage. Using this approach, and working part time, it took our team of four data collectors and analysts, as well as the author, about a year and 4 months to collect and analyze 2,502 speech errors, as shown by the breakdown of the work hours for the different stages below. These estimates can help future researchers plan a similar kind of multi-stage project.

**Table 1:** Stages in creating the database.

Linguistic research Grammar synopsis, reference materials for filling in values, acquiring and organizing lexical resources and corpora	480 h
Training Phonetic training, learning Cantonese phonetic structures, introducing speech errors, listening tests for detecting errors	80 h
Data collection Making speech error submissions with a spreadsheet template	960 h
Data verification and classification Verifying speech errors with reference materials, classifying submissions within taxonomy of speech errors	480 h
Data cleaning Systematizing the data	96 h
Total	2,096 h

### 2.2 Linguistic research

The primary purpose of the linguistic research was to produce a set of reference materials that describe Cantonese language structures in sufficient detail so that these structures can be accurately reflected in the database. To this end, before any speech error data was collected, we created a grammar synopsis that summarized a large body of linguistic facts in a compact “skeletal grammar” of Cantonese (Alderete et al., 2017). The research summarized in the synopsis included primary linguistic descriptions of Cantonese, linguistic research articles, psycholinguistic research, and lexical materials and corpora. The grammar synopsis was necessary because it provided us with a set of linguistic assumptions that supported the analysis of the speech errors. A solid understanding of these structures and how they pattern together is critical to giving an accurate analysis of speech errors. For example, we wished to document whether or not sound errors violated native phonotactic rules (i.e., the rules of legal sound combinations in Cantonese), as this fact is of some theoretical importance (Wells, 1951; Stemberger, 1982a; Alderete and Tupper, 2018). However, in order to classify sound errors as phonologically regular or irregular—that is, whether they obey or violate these rules—it was first necessary to document this rather intricate system of rules in the grammar synopsis.

### 2.3 Training

The goal of this stage was to train data collectors to detect speech errors in the recordings with a reasonable level of accuracy and efficiency. Three of the students that helped write the grammar synopsis were native speakers of Cantonese and continued with the project. We recruited an additional student to create a team with a total of four students (three undergraduate and one MA). All four students were bilingual in English and Cantonese. These students underwent about a month of training, structured as follows. To brush up on phonetic transcription, the trainees read two chapters from a standard phonetics textbook (Ladefoged, 2006). They were then introduced to the phonetic system we adopted to transcribe Cantonese words, which is a slightly modified version of Jyutping (see the sound charts in the grammar synopsis). They were then tested on their transcription skills through two Cantonese dictation tests and given feedback on these tests. All of the students reached a reasonable level of accuracy in phonetic transcription.

The training also involved an introduction to the psycholinguistics of speech errors. This included explaining the standard definition of a speech error (see below), illustrations of valid and invalid errors, and then a review of the basic taxonomy of error types. To make these concepts more concrete, the trainees were then asked to listen to a live conversation outside the lab as a passive observer for an hour and write down as many speech errors as they heard. The idea of this exercise was to illustrate the error types, and, more importantly, to impart on them the fact that speech errors are common experiences in all speakers and not just odd utterances confined to psycholinguistics textbooks.

After this exercise, the trainees were given three listening tests to develop skills in detecting errors through extended practice. In particular, they were given audio recordings of natural conversations in English of approximately 35 min in length that had been pre-screened for errors. Trainees were asked to extract all of the errors in those recordings with a template spreadsheet. They were then given feedback on their submission indicating the errors they correctly detected, the errors they missed, and putative errors that they submitted but that, on closer inspection, do not meet the definition of a speech error. After the three listening tests, all four data collectors achieved a reasonable rate of detecting errors and were allowed to start data collection in earnest.

The listening tests were done in English because we had ready materials for English, and none yet for Cantonese. But the skills acquired in data collection for English clearly transferred to data collection in Cantonese. An alternative approach would be to first develop a set of listening tests in the target language, and then train data collectors with these materials in the target language. However, we do not believe this decision had much impact once data collection had taken off in Cantonese.

### 2.4 Definition of speech error

We employed relatively standard criteria for defining a speech error. A speech error is defined as “an unintended, non-habitual deviation from a speech plan” (Dell, 1986, 284). This definition encompasses sound errors and word errors involving several kinds of manipulations (substitutions, deletions, additions, and shifts), and different directions relating the error and source words (perseverations, anticipations, exchanges, etc.). It also includes word blends and some morpho-syntactic and syntactic errors, like sentence blends and mis-selections of the functional role of a nominal expression. Again, following standard practice, speech errors are not false starts, errors of ignorance, idiolectal or dialectal variants of a word or phrase, or changes of a speech plan, because these are either habitual behaviors or not unintended deviations from a speech plan. Likewise, casual speech phonological patterns are not errors because they are habitual. The reference material from our linguistic research includes a detailed summary of Cantonese casual speech phenomena (Cheung, 1986; Bauer and Benedict, 1997; Matthews and Yip, 2011), and these were frequently used in determining if an utterance is truly an error. One error type that deviates somewhat from prior research is the inclusion of phonetic errors, which we define as correctly selected sounds that are mis-articulated (following Romani et al., 2002). These are distinct from phonological errors, which involve a mis-selection of a discrete sound in phonological encoding. We include phonetic errors because of their increased importance in recent research (Frisch and Wright, 2002; Goldrick and Blumstein, 2006), and on empirical grounds, as our data analysts frequently encounter blended sound errors. While this inclusion may have included some speech errors that would not have been included in prior studies, we think that transcription-based studies may have in fact included many of these phonetic errors as phonological errors because they simply lacked audio back-up for close inspection of the speech.

### 2.5 Data sources

Speech errors were collected exclusively from audio recordings. In particular, the four data collectors listened to roughly 32 h of natural conversations from 50 podcast episodes from third party sources. These recordings came from three different podcast series in which commentators and guests discussed topics from a range of genres, including contemporary film and television, lifestyle, and interpersonal relationships. After reviewing several dozen podcast series, the three series shown below were selected because of their high production quality, long intervals of unscripted speech, and a good balance of speakers for age and gender. The recordings did contain some scripted material, like set introductions and commercials, but speech errors were not collected from these portions because we restricted collection to spontaneous speech. The scripted portions were also excluded in the calculation of total minutes of speech that we used for various data quality metrics discussed below (Table 2).

**Table 2:** Podcast data sources.

情到龍匙 [tsiŋ21dou33luŋ21si2] ‘Master Dragon talks about relationships’ Topics: Relationships and interpersonal problems, feng shui, Chinese horoscope Recordings examined: 13 Total minutes: 492 Database label: ls URL: http://www.sunchiu.com/24773210404084521273.html
電視風雲半世紀 [din22si22fuŋ55wan21bun33sai33gei35] ‘TV dramas in the last five decades’ Topics: Lifestyles and work of guest speakers, members of entertainment industry Recordings examined: 23 Total minutes: 812 Database label: ds URL: http://podcast.rthk.hk/podcast/item_all.php?pid=486&lang=zh-CN
一劇之本 [jat55kek22dzi55bun35] ‘The fundamentals of a script’ Topics: Scripts in theater, movies, TV dramas, cartoons Recordings examined: 14 Total minutes: 613 Database label: yg URL: http://podcast.rthk.hk/podcast/item_all.php?pid=520&lang=zh-CN

While the podcasts reflect different genres, which may in turn affect word frequencies, podcast genre is unlikely to affect the distribution of errors broadly. For example, we examined the percentage occurrence of the linguistic level of the error unit and found that they have similar distributions across all levels: between 84–88% for sound errors, 9–15% for word errors, and close to 1% for both morpheme and syntactic phrase errors. In sum, genre does not seem to affect the error distributions.

Cantonese is spoken in a variety of locations and has many well-known patterns of variation (see Alderete et al., 2017 for review), and so information about the speakers’ linguistic background can help researchers understand the speech error data. In general, the conversations the data were drawn from are rather homogenous linguistically because the three podcasts were recorded in Hong Kong and produced by major Hong Kong producers like Radio Television Hong Kong. All five of the hosts are native speakers of Hong Kong Cantonese. These five hosts contributed 58% of all speech errors, and the remaining errors came from 14 guests on the 電視風雲半世紀 (ds) podcast, and the majority of these are Hong Kong Cantonese speakers as well. The data collection does not have detailed speaker profiles, but the data collectors have recorded over 260 observations about the speech features of individual talkers in the dataRecordings spreadsheet (available on the OSF page, see Appendix). For example, the female host of 情到龍匙 (ls) is slightly younger and has a more casual style exhibiting more “lazy speech” (Matthews and Yip, 2011). This detailed information gives researchers a strong empirical basis to document and analyze language variation in research projects that focus on this variation.

To access the audio recordings for any record in the database, the sound files can be downloaded from the URLs listed above. The File field in the database shows the specific podcast episode. The value for this field uses the following file naming convention: podcastlabelEpisodenumber_airdate. For example, ls021_2012-10-03 = episode 21 of the ls podcast (i.e., 情到龍匙), which has the air date of October 13, 2012. The Time Stamp field (h:mm:ss) in the database indicates the beginning of the first word in the longform of the speech error record. The Podcast field enables searches by podcast (via its label), for example, to search within a specific podcast series.

### 2.6 Data collection

The data collection stage, in which data collectors listen for errors from audio recordings, was the most labor-intensive. Pairs of data collectors were assigned to audio recordings and, like the listening tests, were given a template spreadsheet and asked to document all of the speech errors in the unscripted portions of the conversation. The spreadsheet documented the longform of the speech error and proposed the intended form of the utterance. The spreadsheet also included columns for most of the record fields, including the talker label, time stamp, filename of the audio recording, podcast series, etc. Data collectors listened to the speech with the speech analysis program, Audacity, using high-quality headphones, such as Sony’s dynamic stereo headphones model MDR-7506. With this setup, the data collectors can slow down and re-listen to any stretch of speech, which was often necessary with fast speech. To counteract the natural processes of error correction, data collectors were also instructed to not listen to the recording as a passive observer, as one might listen to a university lecture for example, but instead listen intently to what was actually said in the recording. Data collectors were not given any time limit, and they could listen to a recording as long as they felt necessary. Data collection is also extremely mentally taxing, so breaks were inserted at regular intervals to stay fresh. After tracking several data submissions, we found that successful data collection takes approximately three times as long as the length of the audio recording.

During this stage, detailed notes were also taken for each audio recording in order to document information like the length of the unscripted material, unusual aspects of the conversations, and, importantly, any and all idiosyncratic features of the talkers. We have found that by listening to the same speaker for several hours, and listening intently for speech errors, data collectors become quite adept at spotting these idiosyncratic features because they need to distinguish them from speech errors. These idiosyncratic features are documented as linguistic notes in reference material associated with specific recordings (dataRecordings, see Appendix). This reference material is important to the study of speech errors because idiolectal features are habitual behavior, and so they are not errors. Therefore, it was used initially in data collection, to rule out certain potential errors in the first pass. This reference material is also used in the data verification stage in a similar way, to weed out potential cases that were missed in the initial stage of data collection.

When a recording was completed, a data submission was made with the completed spreadsheet. The database manager then merged the data from the data collectors and imported the speech error data into the larger database for verification and classification.

### 2.7 Verification and classification

Speech errors submitted by the data collectors were processed by the data analyst. The verification stage involved vetting the submitted errors to ensure they are true errors. When submitted errors were deemed true errors by the analyst, they were then classified by filling in all the remaining values for the database fields. These two stages were rather intellectually stimulating, and often required a second opinion. Most of the errors were vetted by a single data analyst, but many of them were done together with another data collector.

Verification involved re-listening to the error and its context, checking it against the reference material on casual speech phenomena and known idiolectal features, and confirming that it meets the definition of a speech error given above. A rule we applied to this stage was that every error in the database should be vetted by someone other than the one who collected it. Thus, for the errors collected by the data analyst, they were confirmed by another member of the team. Typically, the submitted error was first viewed in the longform interface in the Example box, and the analyst tried to form a hypothesis about what kind of error it could be, as well as anticipate potential problems with the error. Then the speech sample is examined to investigate these problems and validate the error. To give a sense of the importance of this step, 3,678 errors were submitted by the data collectors, but only 2,502 errors (68%) were deemed to be true errors. All of the submitted potential errors were retained in the database, but true and spurious errors are distinguished with a record field and justification for excluding a potential error was given. Typical justifications include simple false starts, change of speech plan, casual speech phonology, and idiolectal features. Our verification process tended to be rather conservative, and we generally opted to exclude the speech sample if there is any uncertainty in our exclusion criteria (e.g., is the case a true deviation of the speech plan or simply a change in it?). In Alderete and Davies (2019), we used a similar approach to data verification and analyzed the frequency of different rationales for excluding errors, and found similarly high rates of exclusion. In our study, we concluded that naturalistic speech error corpora require a data verification stage, as corpora that lack such a mechanism exhibit rather different speech error patterns.

The work of classifying errors is essentially a matter of filling in the fields that were not filled in with the initial submission. All of these fields are described in detail on the OSF project page and the motivation for the basic speech error types are given in section 6. In a nutshell, we used a standard taxonomy of speech errors (Dell, 1986; Stemberger, 1993), while introducing several adaptations needed for Chinese languages (Shen, 1993; Wan and Jaeger, 1998; Chen, 1999). Thus, classification requires determining the correct level of linguistic analysis (e.g., sound error vs. word error vs. phrase error) and the type of process that transforms the intended utterance into the error. For example, lexical substitution errors typically involve a substitution (type of process) of a word (level = word). These level/type classes are further distinguished through these major class attributes, like whether the error is contextual (i.e., the source word comes from the linguistic context), as well as special class traits, such as whether or not the error violates phonotactics. Classifying errors often requires resolving or documenting ambiguity (Cutler, 1988), and the processing assumptions (section 5) address this in detail.

### 2.8 Data cleaning

In classifying an error, the data analyst makes several dozen analytical decisions, and some of the classification methods changed in the several months it took to collect and classify speech errors. As a result, the initial classification stage had some inconsistencies, and the data needed to be systematized. To do this cleaning, we used visual analytics software to list all the values of each field and checked them against the established data dependencies and the set of conventions described in sections 4 (linguistic assumptions) and 5 (processing assumptions). Inconsistent values were then corrected, including changes to orthographic conventions, the specific symbols used for Cantonese phonemes, inconsistent syllabic roles, and many missing values. Data cleaning is also a natural outcome of research projects, because by investigating a particular problem, inconsistencies become apparent and new fields become necessary. For example, in writing about tone slips in Alderete et al. (2019), we learned that we had admitted many sequential errors in our database that are better understood as syllable fusions in Chinese languages (see section 4.6). This finding led us to remove some of these blends from the set of true errors in our database. We anticipate that future projects will lead to additional revisions, which will be reflected in future updates.

### 2.9 Internal consistency and data quality

The collection and analysis of speech errors is plagued by problems of data reliability and quality, and problems relating to the perceptual biases of the human listeners that collect the data (Ferber, 1991; Bock, 1996; Pérez et al., 2007). The stages outlined above were designed to address these problems. We believe that the composition of our corpus shows that these methodological decisions have had a positive impact on data quality.

One concern with data quality is whether data collectors detect different types of errors. For example, Ferber (1991) found considerable variation in the types of errors collected from a small sample of German, with one listener detecting no sound errors, and another two detecting no lexical (word) errors. The collection rates for SFUSED Cantonese, broken down by type and collector in Table 3, shows that this is not the case in our collection. Thus, all collectors detected a strong majority of sound errors (averaging 84%) and a non-negligible minority of lexical errors. The error counts are by speaker, but multiple data collectors can report the same error (which occurred roughly 17.5% of the time), so the sum of totals are greater than the 2,502 errors in the database. Collector 3 also submitted far more errors than the other data collectors, and collector 4 much less, but this was due largely to their availability and the recordings assigned to them.

**Table 3:** Error classes by data collector (with overlap and row percentages in parentheses).

	Morpheme	Sound	Lexical	Totals
Collector 1	7 (1.90)	303 (82.11)	59 (15.99)	369
Collector 2	10 (2.09)	409 (85.39)	60 (12.53)	479
Collector 3	25 (1.23)	1,772 (87.20)	235 (11.56)	2032
Collector 4	0	60 (81.08)	14 (18.92)	74

One of the benefits of collecting speech errors from audio recordings is it provides a basis for data validation (Alderete and Davies, 2019). That is, audio recordings enable multiple listeners to collect data, and with these multiple samples, the actual frequency of speech errors in the corpus can be estimated using statistical methods. These estimates can be compared with the errors that were detected, and a percentage of the total of estimated errors can be made. In particular, we use so-called “capture-recapture” methods to estimate error frequency (Chao, 2001). Capture-recapture employs multiple samples of a population, marks individuals (here, speech errors) found in different samples, and then uses the overlap revealed by these marks to estimate the total population. In the data collection stage, each recording was assigned to at least two data collectors, so we have at least two samples for each recording. Following standard practice for heterogeneous datasets like ours, we use formula (14) of Mao et al. (2017) to estimate the lower bound estimate of the frequency of speech errors in the corpus. This analysis indicates that, on average, there was a speech error in our corpus at least as often as every 34 s, or roughly two errors per minute. Our collectors detected 73.84% of these estimated errors. While it is clear that many errors were missed, this sample coverage is approximately 6 or 7 times higher than other data collections made from audio recordings (see Alderete and Davies (2019) for calculations), so we believe it is representative of the kinds of errors that occur in the corpus.

We also report in Table 4 some general measures that speak to data quality. In general, there are far more sound errors (90.05% of the database) than one typically finds in speech error corpora, which Pérez et al. (2007) argue is a feature of corpora with good sample coverage. Likewise, there are large numbers of sound errors that have violations of phonotactic constraints (excluding non-native segments), which may also indicate high data quality (Alderete and Davies, 2019). Finally, there are very low rates of exchanges errors, which are extremely infrequent yet easy to detect, and this fact is consistent with the other measures given here.

**Table 4:** Data quality measures.

Measure	SFUSED Cantonese
Percentage of sound errors	90.05%
Percentage of exchange errors (all types)	0.32%
Percentage of sound errors with phonotactic violations	4.74%
Estimated frequency of errors	At least every 34 s
Percentage of detected errors from estimate	73.84%
Minutes per error (MPE)	0.84

## 3 The structure of longform entries

### 3.1 Orthographic conventions and mark-up

**Figure 1:**
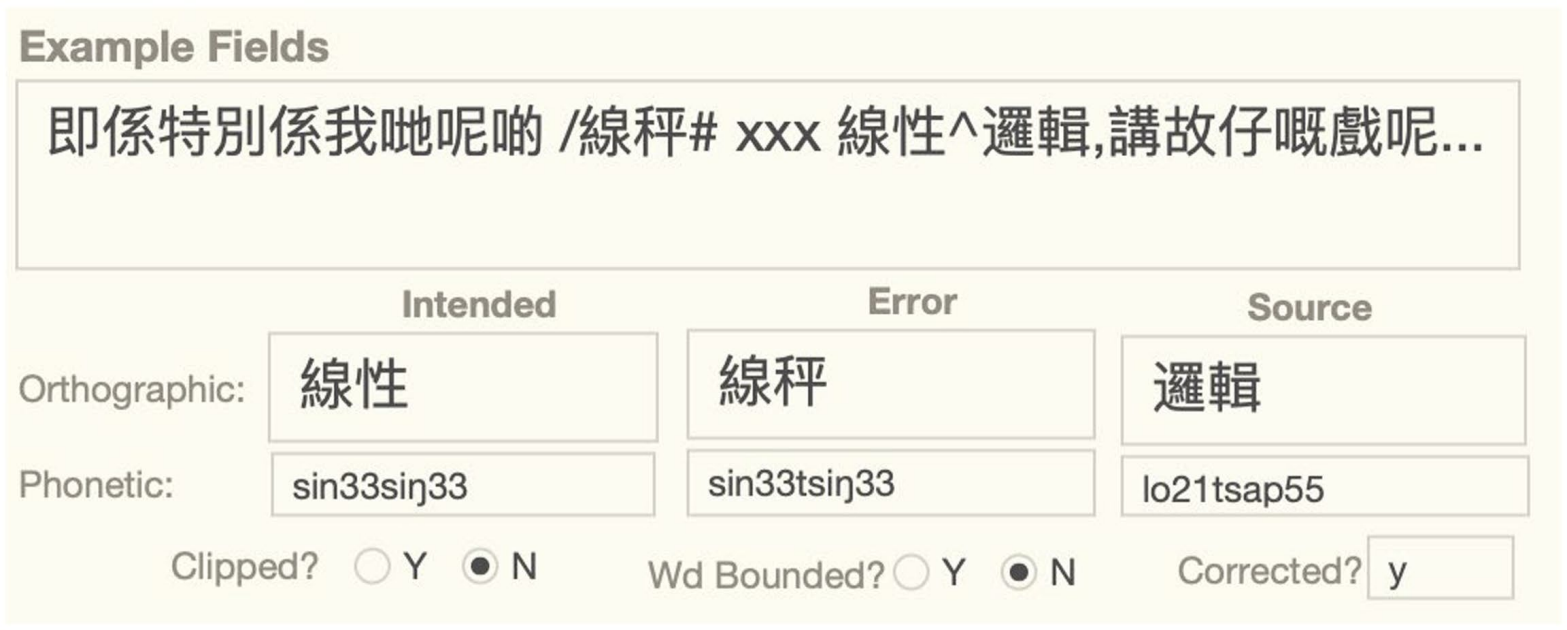
Example box and associated example fields of the phonological substitution error, SFUSED Cantonese 1,289.

The longform of the error is given in the Example box in the example fields section of the longform interface, as shown in Figure 1. It is intended to give an easily readable chunk of the utterance and provide sufficient context to analyze the error. With some exceptions pointed out below, the longform is written in standard Chinese orthography, but annotated to identify the error word with a “/” prefix and potential source words with a “^” prefix. The analysis of the error involves asserting an intended form: if intended form had been used instead, the utterance would not have resulted in an error. Thus, in the example below, we assume that the talker intended to say “線性,” but produced “線秤” instead, with the affricate *ts* replacing the *s* in the second syllable of the intended word. This analysis also assumes that the source for *ts* is the source word “邏輯” that appears downstream of the error. Much of the classification of an error involves assigning attributes to this three-word structure: intended, error, and source. The word and sound fields that appear below the example fields are vertically aligned with these three terms in other field types in the longform interface to allow analysts to associate attributes of the same term.

In general, the longform is written in standard orthography, with long strings of Chinese characters (typically not separated by a space), punctuated with commas and periods (though not the standard period “。” used in everyday Chinese writing). While these standards are used to increase readability, there are important deviations from them to provide more information about the error. These deviations, including the mark-up for the error and source words, are explained in Table 5.

**Table 5:** Mark-up in longform that deviates from standard Chinese spelling.

Mark-up	Usage
/	Prefix used to identify error word or phrase.
^	Prefix used to identify source words; prefixed to the word, not the character or syllable containing the source sound for sound errors. When the source for a sound error is within the error word itself, the error/source word is prefixed with “^/.”
$	Prefix used to identify trigger words in deletion errors (i.e., the ‘source’ for a deletion error, either a sound or a word).
xxx	An interruption in the speech stream (e.g., a pause immediately before an error is corrected).
Empty spaces	In general, spaces are used to segment out important terms and to distinguish words vs. phrases in the intended and error terms. The error word is always preceded and followed by an empty space in the longform, as are “xxx” interruptions. Compounds in the Intended and Error boxes have no spaces inside them, but when an entire phrase is an error, the words inside this phrase are separated by a space, allowing users to distinguish compounds and phrases. The hesitations indicated with commas do not have any spaces. Source words begin with “^” (but no space before) in the longform and are separated from the next word with a space, unless they appear at the end of a sentence or before a comma. When English words are interspersed with Cantonese, there are no spaces between them and Chinese characters in the longform.
#	Right word boundary in polysyllabic error words, i.e., a tool for segmenting out the error word or phrase.
…	Trailing speech at beginning or end of longform (used when it is too cumbersome to write out entire utterance).
,	In addition to normal usage, used to indicate slight hesitations and pauses (which are usually noted in the Notes box).
=	Suffix on clipped error words; used to indicate that the error word or phrase was not completed by the talker; suffix used for all clipped words, including many words and phrases that are not part of the error.
_X=	A gap marker in a clipped word X = where the initial syllable or syllables are omitted.
A:, B:	When the longform of an error involves speaker turns, the utterances of the speakers are introduced with “A:_” for the first talker in the dialog, then “B:_” for the next talker, and then used consistently in the rest of the longform entry.

Occasionally, entries contain more than one error in close proximity to each other. Sometimes these double errors are repeats of the same error word, but they are also often new errors. We generally analyze the first error because of the assumption that the second is more likely to be influenced by the first error than the other way around. The specific error under analysis is apparent from the content of additional example fields (e.g., the intended and error forms). Researchers interested in investigating these double errors can search the longform field for double occurrences of “/,” though these need to be distinguished from two term errors, explained below.

### 3.2 One term vs. two term errors

One term errors are the most common and also the easiest to analyze. A one term error is an error that can be completely described with the replacement of the intended term by the observed error. For example, the example in Figure 1 is a one term error. It can be straightforwardly described as a replacement of the intended 線性 *sin33siŋ33* with the error word 線秤 *sin33tsiŋ33*; the phonological substitution is fully accounted for with this substitution. A little over a dozen speech errors in the corpus (approximately 0.6%) cannot be described this way because they involve more than simple substitution of an intended word for some other word. These involve exchanges and shifts of sounds and words. We call these errors two term errors because they require reference to two terms to give a complete description. Table 6 below lists all of the one term and two term error types in the database.

**Table 6:** One term vs. two term errors.

One term errors	Two term errors
Phonological substitution (non-exchange)	Phonological substitution (exchange)
Phonological addition	Phonological shift
Phonological deletion	Lexical substitution (exchange)
Tone error	Word shift
Phonetic error	
Sequential blend	
Extreme reduction	
Lexical substitution (non-exchange)	
Word addition	
Word deletion	
Word blend	
Role mis-assignment	
Sentence blend	
Morphological error	
Complex set of processes (usually)	

**Figure 2:**
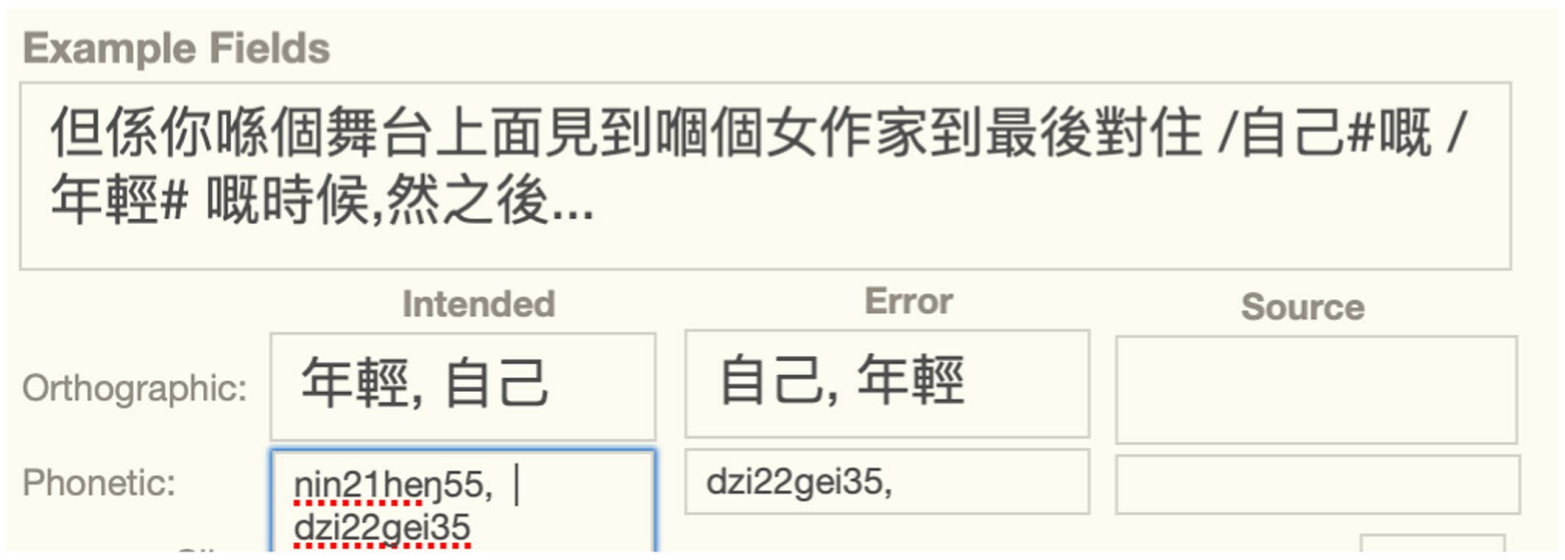
Lexical substitution exchange containing two error positions and word pairs.

Exchanges cannot be described as simple substitutions because there is a substitution with a word downstream simultaneously with perseveration of an upstream word. In the lexical substitution exchange error below, both words in the error are intended, but they are also both error words because they occur in the wrong position. To describe them in the longform, we need two terms prefixed with “/,” as illustrated in the longform of the example below involving a lexical substitution exchange (Figure 2).

**Figure 3:**
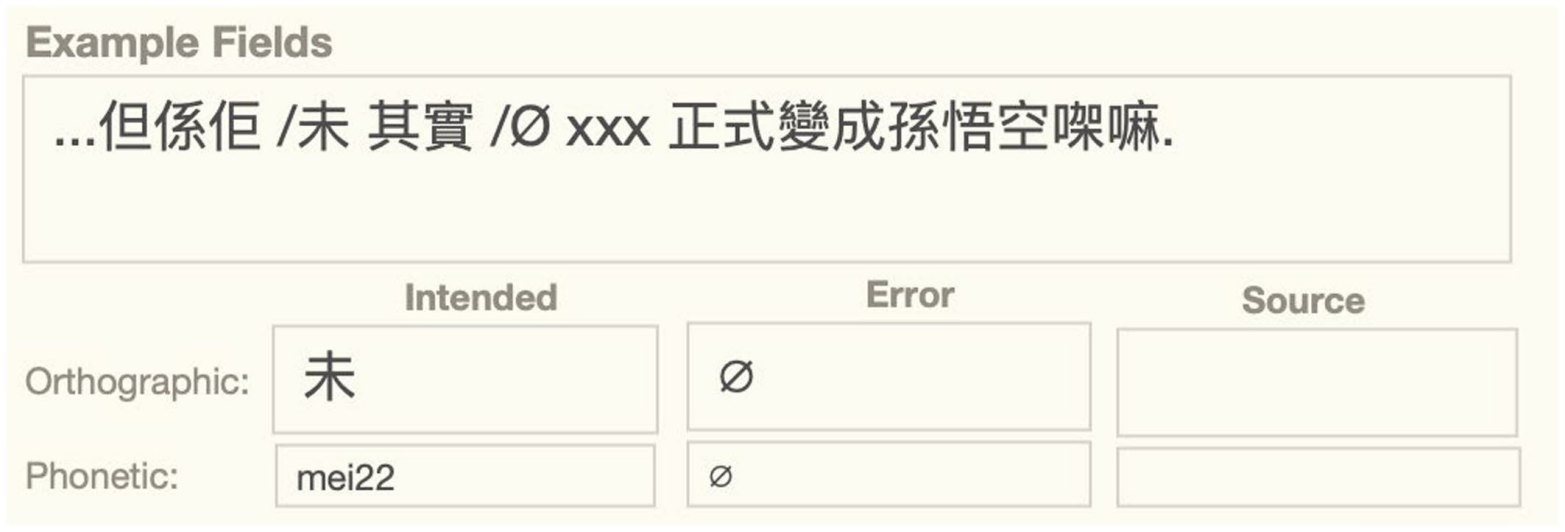
Word shift error showing both shifted word and its origin, SFUSED Cantonese 1971.

Likewise, shift errors involve an error word in some illicit position, but the analysis of the error also requires an assumption about the correct position for the shifted word, which requires a second term. In word shifts, for example, there is an error in the position of the shifted word, as well as a word omission error in its intended slot. As shown below, we use the null symbol “Ø” to mark the licit position for a shifted word (Figure 3).

Note that word blends, though they seem to involve two intended terms, are not two term errors in this sense because there is only a single slot for the error. They can be described as a single substitution of a word pair with the blended error term. Thus, the English blend *traith* from SFUSED English 1,471 (the record ID number here and throughout can be used to look up speech errors in SFUSED) can be analyzed by replacing the error word with the word pair (*trust, faith*), which is a coherent term. The distinction between one term and two term errors is essentially an technical issue for representing the data structure, and it is not intended to carry with it any special processing assumptions. We are principally interested in getting the nuts and bolts of the coding of errors in a robust system, so that researchers can search the longform entry in useful ways.

### 3.3 Contextual sensitivity

The distinction between one and two term errors, as well as some other quirks of certain other error types, like blends, requires some contextual sensitivity in interpreting some of the fields in the database. That is, we have developed a robust and intuitive interface for one term errors, but to extend them to two term errors and word pairs would require adding an unnecessary amount of complexity to the database. Instead of creating this complexity, we have opted to interpret the field values for certain error types with contextual sensitivity. That is, we give special meanings to the fields in specific contexts. These special meanings are given below, broken down by master type (Table 7).

**Table 7:** Assumptions about context sensitivity, by master type.

Master type	Contextual sensitivity
Exchanges	The two terms both have the prefix “/” in the longform. The Intended fields contain the two intended terms in the correct order, and the Error fields contain the two error words in the observed order.
Phonological shift	The two terms both have the prefix “/” in the longform. The Intended fields contain a word pair of the two intended words, and the Error fields contain the two error words.
Word shift	The shifted word and “Ø” appear wherever they appear in the example, but the shifted word is given in Intended, and “Ø” in the Error field.
Word blends	Intended contains a pair of words.

Finally, both one term and two term errors allow terms to be pairs of units. This complicates the assignment of other attributions, like part of speech labels and open/closed category status because both units need an attribute. However, in most cases, the two units have the same attribute, so the specified value of that field applies to both units. In the small number of cases where this is not the case, the two values are specified in Notes.

## 4 Linguistic assumptions

The coding of speech errors requires linguistic assumptions. For example, the word fields require a consistent set of part of speech categories, and whether or not a sound error is phonologically illicit or not requires reference to a coherent system of phonotactics. Many of these assumptions rely on the grammatical structures that are explained in our grammatical synopsis (Alderete et al., 2017) or studies referred to in that document. However, we give a crisp summary of these structures here to provide a linguistic background for the logic of the database and the processing assumptions.

### 4.1 Phonetic transcription

An adapted version of the International Phonetic Alphabet (IPA) is used to transcribe consonants and vowels. Within this system of phonetic symbols, the contrast between aspirated vs. unaspirated sounds are transcribed as voiceless vs. voiced sounds, consistent with Jyutping and Yale romanization conventions. However, we make some small changes to these romanizations, such as IPA *ŋ* for the velar nasal instead of the digraph *ng* in Jyutping and Yale. For a full list of phonetic symbols, and their counterparts in other phonetic systems, including Jyutping, please see the Appendix of our grammar synopsis (Alderete et al., 2017). Phonetic transcription is used throughout the sound fields and the example fields for phonetic words and phrases. Though the longform entry in the example box uses Chinese characters whenever possible, words are occasionally written in phonetic symbols in cases where there is no standard spelling, as in the error word */jip35* for intended word 咦 *ji35* (SFUSED Cantonese 440).

Most sound errors are transcribed with this system. However, phonetic errors, or gradient errors, require us to adapt this system because they involve phonetic structures that are not part of the standard sound inventory. We define phonetic errors as sound errors that involve the selection of the correct sounds in phonological encoding but are mis-articulated in phonetic processing. Drawing on research on gradience in psycholinguistics (Stoel-Gammon, 2001; Frisch and Wright, 2002; Goldrick and Blumstein, 2006), we recognize three basic types in SFUSED Cantonese. Ambiguous phonetic errors that fall between two sounds (transcribed as A|B, for two sounds A and B), transitional errors that move from one sound to another (A-B), and non-native tones that have a similar transition or ambiguous status. For example, the ambiguous phonetic error *bœ|oŋ55* (SFUSED Cantonese 209) contains an unchanging vowel that is somewhere between *œ* and *o*, and the transitional phonetic error *laːm21n-lan35* (SFUSED Cantonese 137), has an onset that transitions between *n* and *l*.

### 4.2 Tone

Table 8 below exemplifies the tones in Cantonese, and shows the Chao digits we used to transcribe tone (Chao, 1930). Modern Hong Kong Cantonese has six basic tones, which are contrastive in height (high, mid, and low) and contour (level, rising, and falling). The three level tones in Cantonese have allotones, which are known as checked tones. These tones are limited to syllables ending in an unreleased stop and are shorter in duration than their non-checked counterparts. We do not represent these allotones in our sound fields because they are predictable, and standard practice in phonological analysis is that they represent the same phonological tone (Yue-Hashimoto, 1972).

**Table 8:** The six tones of Cantonese (Matthews and Yip, 2011, 27).

High level	55	憂 jau55 ‘worry’
High rising	35	油 jau35 ‘paint’
Mid level	33	幼 jau33 ‘thin’
Low falling	21	油 jau21 ‘oil’
Low rising	23	有 jau23 ‘have’
Low level	22	又 jau22 ‘again’

The high level [55] tone is contrastive with an additional tone, namely the high falling [53] tone, in Guangzhou dialects. Furthermore, [55] and [53] are in free variation in the speech of some older speakers of Hong Kong Cantonese, but it is neutralized to [55] for most young speakers (Bauer and Benedict, 1997). The [55/53] distinction is only occasionally observed in our corpus of speech errors, and observations are made in the Notes box when it is important to document the error. Cantonese also has a change in progress involving a set of tone mergers that is important to documenting tone errors. Acoustic studies of the Hong Kong Cantonese tones have revealed that some speakers do not always discriminate between certain tone pairs in perception and/or production, notably between the rising tones [35] and [23], the level tones [33] and [22], and between tones [21] and [22] (Bauer et al., 2003; Mok et al., 2013). The data analysts were aware of these mergers when they initially classified the tone errors, because of their potential to be confused with actual tone errors. Additionally, in a later systematization stage, all tone errors were re-examined by two data analysts to confirm that tone mergers were not mistaken as true tone errors. During this stage a handful of cases were excluded from the database. It is possible that a small number of tone errors in the database are in fact the results of these mergers, but we think this is unlikely for two reasons. Many tone errors involving substitutions of the tones involved in these mergers were actually corrected, reflecting the fact that these tones are distinct tones in the minds of our speakers. Furthermore, if these tone slips are in fact due to tone mergers, we would expect far higher numbers of slips than were actually observed. This is because five of the six tones participate in the mergers, and so, if mergers were indeed misheard as errors, there would have been an opportunity for such a mistake in classifying errors in almost every word.

Tone production is inherently variable and difficult to ascertain in some cases. In general, somewhat odd tones in speech errors that were not perfect exemplars of a tone category were counted as standard phonological errors in two conditions: if the data analyst judged that the tone was in the normal range of variation (and so a valid exemplar of a given tone), or if the odd tone could have arisen from co-articulation with a following or preceding tone. However, if an abnormal tone lacked one of these properties, they were treated as a phonetic blend of two categories or a phonologically illegal tone substitution error and are labeled as such in the sound fields.

### 4.3 Part of speech categories

Part of speech categories are important information specified in the word fields. We recognize the following 18 categories, briefly described below and explained in more detail in section 4 of the Cantonese grammar synopsis (Alderete et al., 2017). Errors consisting of coherent phrases are assigned a part of speech determined by the head of the phrase. An error involving a string of words that is not a constituent is described as a string of the categories in the Notes box. The following part of speech categories are open class categories in Cantonese: Adjective, Adverb, Name, Noun, and Verb. All other categories are closed class, as indicated at Table 9.

**Table 9:** Part of speech categories.

Category	Description
Noun (open)	Head of a noun phrase
Verb (open)	Head of a verb phrase
Adjective (open)	Describes the qualities or characteristics of nouns
Adverb (open)	Qualifies verbs, describing the manner of an action or its circumstances
Name (open)	Proper name
Coverb (closed)	Grammatical morpheme that functions as a preposition, but also exhibits properties typical of verbs; for example, coverbs can take an aspect marker or verbal particle; coverbs may occur without a following verb, but the coverb and its arguments usually modify the verb phrase it follows
Localizer (closed)	Grammatical expressions that either function as adverbs of location or postpositions
Pronoun (closed)	Deictic expression marked for person, number (with suffix *-dei22* for plural); 3sg pronoun *kœi23* can replace animate, inanimate, and abstract entities
Determiner (closed)	Deictic expression that picks out objects in time and space, including demonstratives *this*, *that*, *here*, and *then* and numerals
Classifier (closed)	Obligatory grammatical morpheme occurring before the noun, serving to classify nouns loosely based on shape, natural kind, and function; two or more alternative classifiers are available for some nouns
Auxiliary (closed)	Function word appearing before the first verb and functions similarly to other verbs; an auxiliary does not take an aspect marker or verb particle, and it does not carry tense, aspect or mood; when the main verb is clear, the verb can be dropped with auxiliary support
Conjunction (closed)	Grammatical morpheme that conjoins two words or phrases of the same class; can be overt or null
Sentence final particle (closed)	Grammatical morpheme at the end of a topic or a clause and serves three major pragmatic functions: indicating speech-act types, conveying evidentiality, and emotional coloring
Verbal particle (closed)	Grammatical morpheme following the main verb and indicates results, direction, adversaries, or habits
Aspect marker (closed)	Bound form that marks aspect and behaves essentially like suffixes; it cannot be separated from a verb
Negator (closed)	Morphemes *m21* and *mou23*; the former precedes a verb for negative imperative or present or future verbal negation; the latter precedes a verb for negation of past events
Linker GE (closed)	Grammatical morpheme linking modifying expressions and head nouns; may be omitted in certain environments, including with kinship terms and other nouns with a close link between the possessor and the noun
Interjection (closed)	Simple emotive or filler word

### 4.4 Compound vs. phrase

In order to document the intended, error, and source terms accurately, we needed to distinguish compounds vs. phrases. Compounds are single words that are formed by combining two (or more) free words. So-called compositions are also single words but involve combining at least one bound root with another root. Compounds and compositions (which are not distinguished in the longform entries) contrast with phrases, which are constructions composed of two or more words. Because the word fields require this distinction, and language processing is also sensitive to the distinction between words and phrases, we distinguish them by inserting a space between the words of a phrase, but no space inside of compounds (see section 3.1 for more details on orthographic conventions).

It is not always obvious how to distinguish compounds from phrases in sentences, especially in Verb + Noun sequences, so we use two standard tools for distinguishing them (Matthews and Yip, 2011). First, compounds may have an idiomatic meaning that is not predictable from its parts, as in, 飲茶 *jam35tsa:21* ‘have dim sum’ (literally, ‘drink-tea’), whereas phrases generally do not have these non-transparent meanings. Second, when an aspect marker or verbal particle is inserted between the verb and object, the resulting meaning tends to correspond to an intransive verb in English and the meaning of the object is lost, as in, 皺眉頭*dzau33mei21tau21* ‘frown’ (literally, ‘wrinkle-eyebrow’), so this is, again, a test for compounds.

### 4.5 Cantonese syllable structure and phonotactics

Knowledge of Cantonese syllables is necessary to understand the values in the sound fields, and knowledge of Cantonese phonotactics is necessary to know if a speech error is phonologically illicit. We sketch the syllable structure below, and the system of phonotactics based on it, following standard assumptions in Cantonese phonology (Yue-Hashimoto, 1972; Cheung, 1986; Bauer and Benedict, 1997; Pulleyblank, 1997).

Cantonese syllables typically have the form (C_1_) V_1_ (X_2_), allowing a single consonant in onset (C_1_) and coda (X_2_) positions. In general, any consonant, including the semivowels *j* and *w*, may appear in onset position. Also, though *ʔ* is not counted as a phoneme of Cantonese, it may appear in onset position as an optional variant of an empty onset (as in, *au35 → ʔau35* ‘to vomit’) or *ŋ* (e.g., *ŋaːu21 → ʔaːu21* ‘cow’). However, the coda and rime are more restricted, as are tone + syllable combinations. These restrictions are explained in Table 10, illustrated in Table 11, and cross-classified by syllabic role in the special class field Phonotactic Violation.

**Table 10:** Cantonese coda and rime phonotactics.

**Coda:** (C_2_)
C_2_ can be filled by any of /m n ŋ p t k/
The combination of a nucleus and a coda is restricted in Cantonese in the rime inventory (see Table 11)
**Rime:** V_1_ (X_2_)
V_1_ in an open syllable can be filled by any member of the set /i e y œ aː o u/, but not the short vowel *a*
All eight vowels in V_1_ can combine with a non-identical high back vowel, either *i* or *u*, in X_2_ position to form one of 11 diphthongs (see below), but these diphthongs cannot occur with a coda consonant, which also occupies X_2_
Vowels can sometimes be reduced to schwa *ə* due to the casual speech phenomenon
V_1_ can be filled by two syllabic consonants, *ŋ* and *m*, which cannot combine with a coda consonant (i.e., the syllabic consonants fill the entire rime)
Vowels in V_1_ do not freely combine with all possible coda consonants in X_2_ position, as illustrated in Table 11
**Tone**:
Each syllable carries one tone (see legal tones in 4.2)
So-called checked syllables ending in unreleased /p t k/ cannot carry a contour tone (i.e., tones 53 23 21)
Tone 53 is licit for speakers with 53 ~ 55 free variation (generally true of older speakers), and illicit otherwise

**Table 11:** Cantonese rimes.

	i	e	y	œ	a	aː	o	u	ŋ	m
N									ŋ	m
V	i	e	y	œ		a:	o	u		
V + i		ei		œi	ai	aːi	oi	ui		
V + u	iu	(eu)			au	aːu	ou			
V + m/p	im	(em)			am	aːm				
V + n/t	in	(en)	yn	œn	an	aːn	on	un		
V + ŋ/k	iŋ	eŋ		œŋ	aŋ	aːŋ	oŋ	uŋ		

### 4.6 Extreme reductions, sequential blends, and syllable fusion

Certain errors, and also some habitual behaviors, involve a radical reduction of a string of segments, as illustrated in Table 12. Sequential blends, like *Tennedy* for *Ted Kennedy* in English, involve deletion of certain portions of two adjacent strings. These blends can resemble so-called “extreme reductions,” as in [ætʃi] for *actually*, in that in both cases, multiple segments are deleted. They differ, however, in that sequential blends tend to have fully articulated vowels and consonants and are usually triggered by similar or identical segments in the two strings, as in [ɛ] in *Tennedy* (Shattuck-Hufnagel, 1979). Extreme reductions are not restricted in these ways and may combine deletions of whole segments with reduced segments that fail to meet their articulatory targets (Ernestus and Warner, 2011). In addition, Chinese languages have a set of canonical syllable reduction patterns, sometimes called syllable fusion, that remove rimes and sometimes onsets in bisyllabic sequences in predictable ways (Cheung, 1986; Wong, 2006). These can resemble extreme reductions and sequential blends because they also remove long strings of segments in adjacent morphemes. To distinguish these phenomena, we first examined each case to see if they fit the specific reduction patterns that have been established for Cantonese (see Alderete et al., 2017, 16 for the five basic patterns). If they did match these patterns, they were not counted as errors because they are habitual. While extreme reductions can, in principle, be characterized as habitual activities, and therefore do not meet the definition of being an error, we include extreme reductions in the database because they are difficult to distinguish from sequential blends and are interesting in their own right. Following standard practice, sequential blends are included in the database and counted as errors.

**Table 12:** Reduction phenomena.

Sequential blends (are errors)	/sœn21sœi23 hai22 / *→* sœn22 hœi22 純粹係 ‘it solely is’ (SFUSED Cantonese 1,585)
Extreme reductions (not errors, but included)	/gam55dzaːp22/ *→* gaː55ə22 今集 ‘this episode’ (SFUSED Cantonese 559)
Syllable fusion (not errors, not included)	/faːn55 di55/ *→* faːi55 返啲 ‘do it again’ (SFUSED Cantonese 242)

## 5 Processing assumptions

A number of processing assumptions are necessary to classify speech errors. In general, we have assumed an activation dynamics model as an underlying system for explaining most speech errors, a consensus view in the language production literature (Stemberger, 1985; Dell, 1986; Levelt et al., 1999; Goldrick, 2007). For example, in a sound anticipation like /reading list/ *→ [l]eading list*, we assume that anticipatory activation of a downstream *l* has supplanted the production of the intended segment *r.* Our core classification of errors, however, does not require an activation dynamics as an underlying architecture. The values for all the fields can be interpreted as simple descriptions of the speech error itself. There are, however, specific assumptions we must make in order to complete the fields of the database, and we explain them here so that future research will be aware that these are just assumptions.

### 5.1 Master types

Master types are convenient categories for speech errors that simplify data input and searches. In addition, they are intended to reflect our understanding of language production processes in contemporary language production research. Thus, the four basic phonological errors (i.e., substitutions, additions, deletions, and shifts) correspond to the established error patterns in phonological encoding, or errors of selecting sounds in speech planning (Shattuck-Hufnagel, 1979; Stemberger, 1993). To this class, we can add tone errors, because mis-selections of tone are rather common and most likely errors of phonological encoding (Wan and Jaeger, 1998; Alderete et al., 2019). Sequential blends are also errors of phonological encoding because they involve sublexical mis-selection of segments (Laubstein, 1999). Phonetic errors are assumed to be correctly selected sounds that are mis-articulated, and thus part of articulatory processing (Frisch and Wright, 2002; Goldrick and Blumstein, 2006). Word level errors can also be cross-classified by using established production processes. Thus, lexical substitution errors are errors of lexical selection, and role mis-selections are errors of function assignment, or the linking of entities to the logical arguments of a sentence (Bock and Levelt, 1994). While they are perhaps less well-understood, we also assume that word errors such as word additions, deletions, and shifts are also errors of grammatical encoding, as are sentence blends and morphological errors (Stemberger, 1982b). For descriptive power, we also include two additional master types: Complex Set of Processes, which can combine any basic error type, and One of a Kind, which is any error that does not fit these known categories but nonetheless meets the definition of a speech error. In sum, master types are convenient labels that correspond to errors with established production processes.

### 5.2 Ambiguous errors

**Figure 4:**
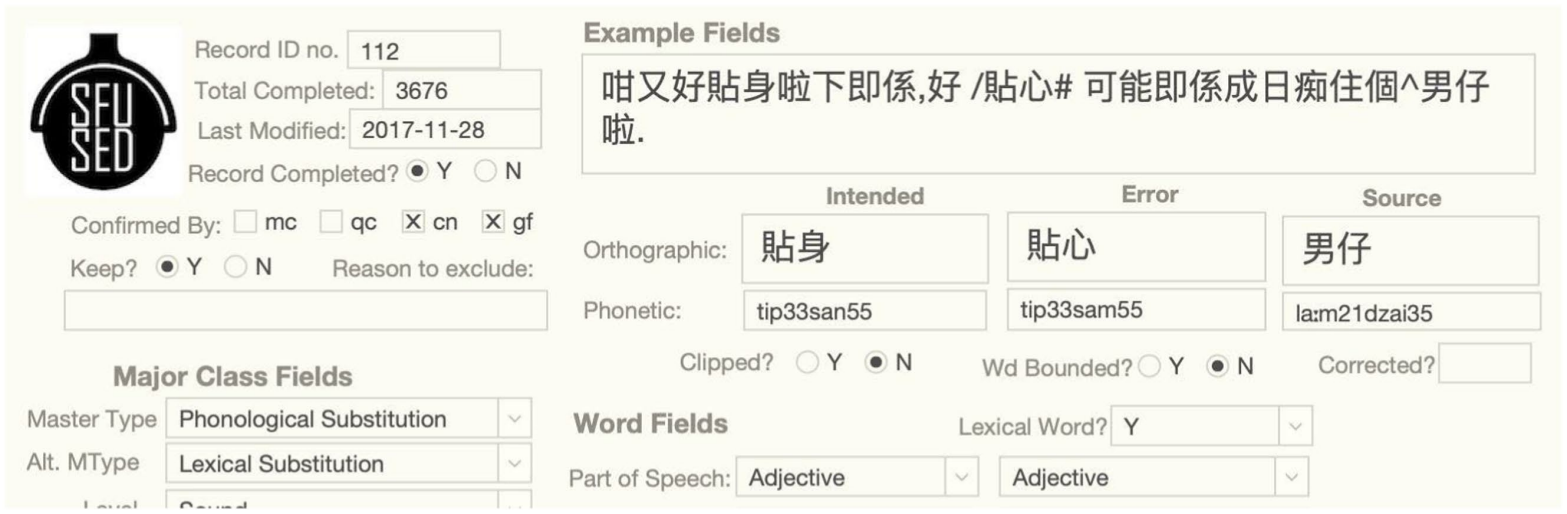
Illustration of ambiguity.

Many errors are ambiguous in the sense that they can be classified as more than one master type. For example, there are several phonological substitutions that could also be lexical substitutions, because the substitution of the sound produces an actual word, and so the error could instead be a mis-selected word containing most of the target sounds. For example, swapping *m* for *n* in the speech error in Figure 4 produces a new word, which makes possible the substitution of the intended 貼身 for the error word 貼心. In cases such as these, the proposed master type is the most plausible, given the available evidence, and the alternative master type is given in the Alternative Master Type field, as shown below. Our practice with such ambiguous cases is to seriously consider both possibilities, assemble the evidence pointing in favor of both of them, and then pick the analysis with the most evidence, similar to the strategy advocated in Stemberger (1982/1985). This kind of ambiguity is rare, but most common between phonological errors and lexical substitutions, as well as tone errors and lexical substitutions. In some cases, however, the sentential context does not support a word substitution semantically, so we assume there is no ambiguity and no alternate master type is given.

### 5.3 Error context: sources, triggers, directionality, and source ambiguity

A basic assumption of contemporary approaches to speech errors is that many speech errors are contextual in the sense that attention to a nearby word produces an error. In models with activation dynamics, speech errors arise because a nearby word or sound receives higher overall activation through this attention than the intended word, causing it to supplant the intended. We implement this idea using relatively standard characterizations of source and trigger units and their directionality (Shattuck-Hufnagel, 1979; Dell, 1986; Stemberger, 1993). Thus, source units (e.g., words or sounds) are linguistic units that are identical to the intruding unit in a contextual error. Trigger units, a term due to Shattuck-Hufnagel (1979), are linguistic units in the context that are identical to the unit deleted in deletion errors. Following the SFUSED typographic conventions (see section 3), source units are prefixed with “^” and triggers with “$.”

What is the window in which these contextual units can interact with other words to produce errors? Though we are aware of certain windows employed in the literature (Nooteboom, 1969), we have taken a conservative approach in positing a rather large window of 10 syllables upstream and downstream of the error locus. This 20-syllable envelope excludes repetitions or recasts after an error because these are part of the original speech plan. Our somewhat wider window (*cf.* Nooteboom, 1969 six syllable window in both directions, or a 12-syllable envelope), is motivated by the belief that many examples seem to require a wider context, for example, with highly salient words. More importantly, however, we believe that the exact nature of this window is not yet well understood, and that many unexplored factors, including the chance occurrence of source sounds, are likely to be crucial in the analysis of whether a nearby word or sound leads to an error. A wider window therefore allows for these different factors to be explored in later work. As an example, in our project on tone errors (Alderete et al., 2019), we also reclassified the data for the Direction field based on two different windows (i.e., four and seven syllables).

Source and trigger units have a directionality, or a position relative to the error word. Following the system in Stemberger (1993), we recognize the standard categories of perseveration, anticipation, broken anticipation (a.k.a. incompletes, which have a break between the error and downstream source or trigger unit), exchanges, and combined perseveration and anticipations. These categories are specified in the Direction field and determine if the error is contextual or not.

Often there are more than one source unit or trigger. This is not a problem for the Direction field, because the coding scheme allows for multiple source and trigger units. However, in order to fully analyze sound errors, we must identify the attributes of a single source word. Our basic strategy is to include all source and trigger words in the longform of the entry but try to identify the most likely contextual unit for the sound fields, which is stated as an assumption in the Notes box. Thus, we assume that units are more likely to be sources if they are closer to the error, match in syllable role and word position, and have identical neighboring segments (MacKay, 1970; Dell, 1984). This selection procedure may have the effect of skewing patterns toward established speech error patterns, so care should be taken in evaluating these patterns and comparing them with other languages. However, the true complexity of the contextual units is accurately represented in the longform of the errors, so future research can build more complex analyses from these rich contexts.

### 5.4 Lexicality and clipped words

Sound errors can lead to actual words or nonsense words. In general, we have relied on the native speaker intuitions of the data analysis in determining when a sound error produces a word of Cantonese. This is usually a simple mental search by the data analyst to assess the wordhood of an error, but occasionally, if the data analysis is unsure, lexical resources and the internet are used to test wordhood. We believe this is a reasonable approach to assessing the lexicality of sound errors because the impact of the lexicon in producing actual words is surely the mental lexicon of the talkers in our audio recordings, not an exhaustive lexical resource.

Many lexical words are clipped before they are completed by the talker. For sound errors, it is not possible to ascertain the lexicality of the error because we simply do not know how it would have been completed. With clipped sound errors, therefore, we consider what the word would have been if the error word had been completed as intended, mis-selected segments aside. If the error would have resulted in an actual word if the unuttered portion was completed as intended, Lexical Word? = “Likely Y,” and “Likely N” otherwise.

### 5.5 Semantic relationships

It is well-known that lexical substitutions tend to be semantically related in the sense that intended and error words share certain semantic features or properties (Harley and MacAndrew, 2001; Jaeger, 2005). The Error-Intended Semantic Relationship field is intended as a first approximation of the nature of these relationships, based on prior research and certain facts we observe in the database. An important principle is that intended and error words are related in all speech errors in some concrete way, and so this field is important in cross-classifying all errors.

Starting with phonological and phonetic errors, a basic assumption is that the error word is a botched attempt at the same lexeme as the intended word. This is because modern psycholinguistics assumes that the right word has been selected in sound errors, but there has been an error in phonological or phonetic encoding (Bock and Levelt, 1994). Thus, all sound errors have the semantic relationship of “Same meaning (same lexeme).” Likewise with other sublexical errors, such as sequential blends, extreme reductions, and tone errors, we assume that the correct lexeme has been selected in lexical selection, and the error occurred in a downstream process.

Word level errors, on the other hand, have intended-error pairs whose members are not the same in meaning. Perhaps the easiest relationship to grasp is “Same semantic field,” which is a common relationship in which the two words share many attributes and tend to be used in the same contexts, like the words *broccoli* and *cauliflower* (sometimes dubbed “co-hyponyms” or “coordinates”). Word pairs can also be related via the ““Goes with” (thematically related)” relation, like the words *spider* and *web*. Finally, we have found a number of self-explanatory relationships to be necessary in describing the observed errors, including “Same meaning, different part of speech,” “Antonyms,” “Synonyms (but not same lexeme),” and “Near-Synonyms.” Word errors also may not have an obvious relationship, in which case they are specified “Not obviously related.” The same semantic relationships that apply to words may also apply to phrases, as long as they have the characteristics described above.

Word additions, word deletions, and word shifts do not have a semantic relationship between intended and error words because they do not have both of these terms, so they are specified “NotAppl.” The database also specifies a semantic relationship for word and sentence blends, but this field is context-sensitive in the sense that blends are evaluated for the two parts of the blend, rather than the error and intended word. Thus, it has been shown that blends also have interesting semantic relationships that overlap with those of lexical selections (Fromkin, 1971), and we re-use the semantic relationships value list for characterizing this in blends.

## 6 Using the two interfaces

There are two basic ways to examine the speech error data, the longform interface that allows one to drill down into the facts of a single speech error, and the dimensions interface that supports investigations of the entire corpus. The discussion below anticipates some of the more common uses of speech error data, links them to existing projects, and sketches ways of exploring the raw data frames in other frameworks.

### 6.1 The longform interface

**Figure 5:**
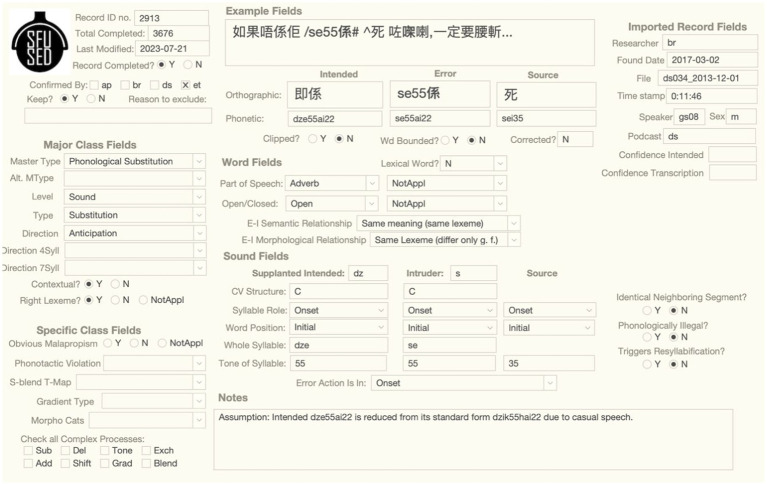
Longform interface for the sound error.

The longform interface presents all of the information about a speech error in a single screen, as shown in Figure 5 for the sound error /dze55ai22/ → *se55ai22*. Fields of the same type are clustered together. For example, the example fields documenting the full context and other descriptive aspects of the error are center-top, and the major and special class fields are on the left. The layout of the center portion of the interface analyzes the error into Intended, Error, and Source forms (if there is a source form). Importantly, these data types are aligned for the example fields, word fields, and sound fields, so researchers can track the same information vertically. For example, the error word is in the middle column in the example fields and the Intruder (=error) sound in the sound field is aligned with the error word near the bottom of the screen.

The longform interface is best for data input and classification, viewing illustrations, and any research that involves drilling down into the details of a particular example. For reference, the Notes box at the bottom states any assumptions the researchers have made in classifying an error, and the Time Stamp and File fields on the right give the information needed to listen to the audio recording. Users can look up examples referred to in a journal article by searching for the number in the Record ID no. field.

This interface can be searched with limited search tools, for example, compiling a list of all phonological substitutions or word blends, and the results of these searches, or the entire database, can be exported to a spreadsheet. However, more powerful search tools for research purposes are available in the dimensions interface. Users can work in the longform interface using software programs like FileMakerPro and MySQL. The OSF data release includes a longform interface created with FileMakerPro, as well as the raw data in a CSV file that can be imported into any database program.

### 6.2 The dimensions interface

Research on speech errors is often interested in finding speech error patterns in the entire data set, or building contingency tables that show the distributions of errors cross-classified by certain conditions. The dimensions interface is a more effective way of conducting such research because it gives a bird’s eye view of the database, rather than drilling down into the facts of a specific entry. The dimensions interface can be explored with a variety of software products, like Tableau, Google Data Studio, R, and Python pandas.

**Figure 6:**
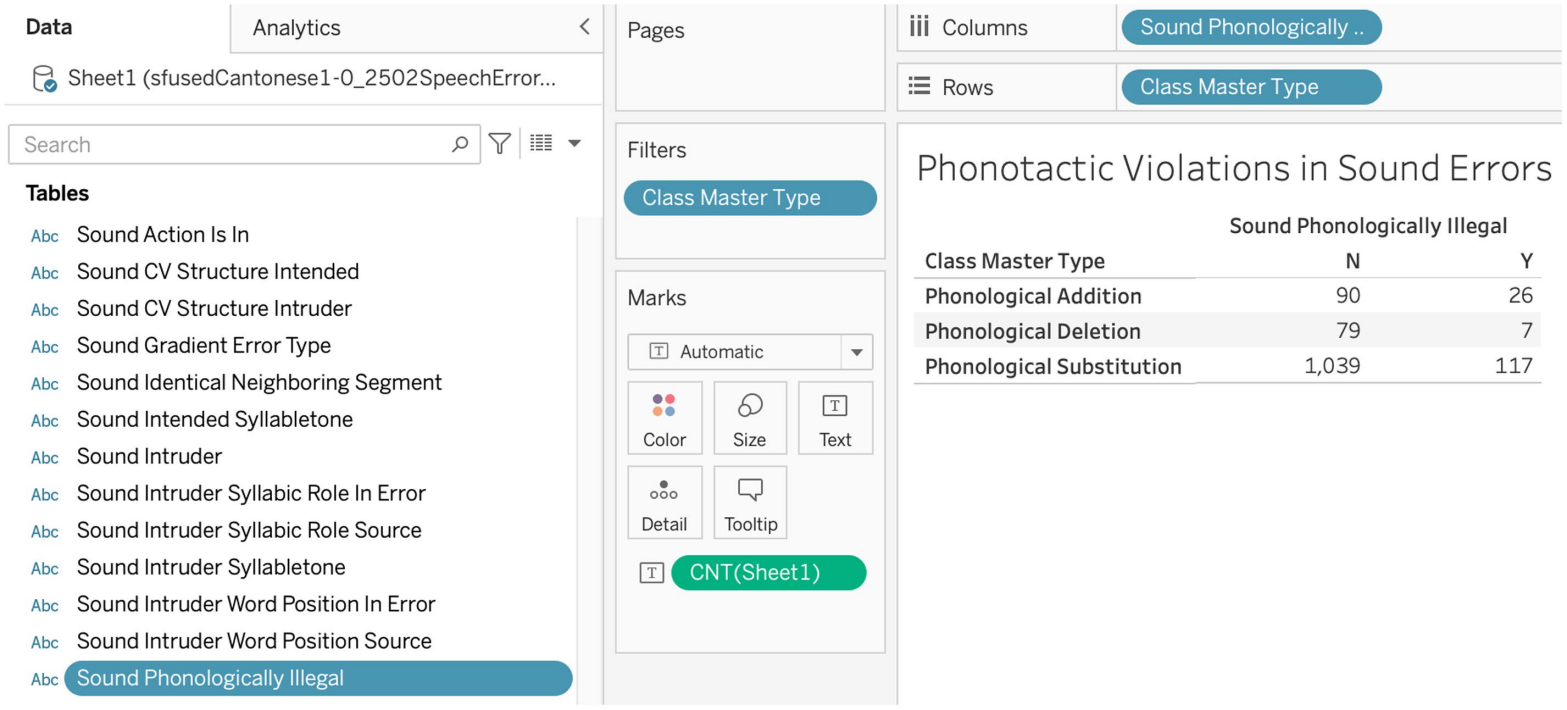
A contingency table showing phonotactic violations in sound errors.

The figures below illustrate the functionality of the dimensions interface with two kinds of common data tables in Tableau. Figure 6 shows how the incidence of phonotactic violations can be cross-tabulated by the three core master types for sound errors in a contingency table, first reported in Alderete and Tupper (2018). Here, we are interested in counts, so the center cells have been specified for the measure Number of Records. The rows are specified for the major class field, Master Type, which has been filtered to include only these three core types of phonological errors. The column is specified for the sound field Sound Phonologically Illegal, which simply cross-classifies errors by whether they violate phonotactics (Phonologically Illegal = Y) or not (N). In all of these data science platforms, the data can be extracted as cross-tabulated tables for further analysis.

**Figure 7:**
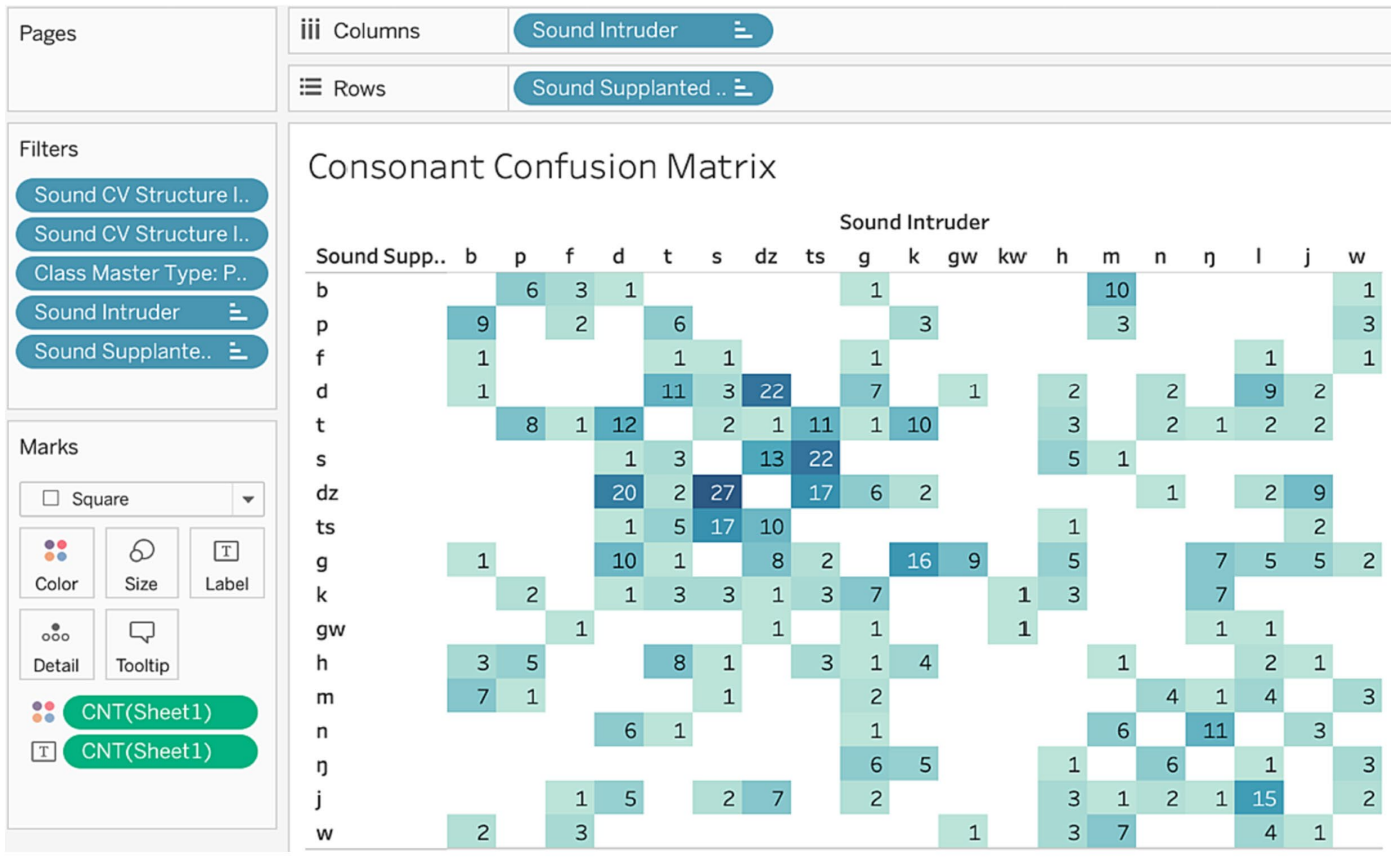
Consonant confusion matrix.

Another type of table commonly used in speech error research is a confusion matrix, showing how intended structures are confused in errors. Figure 7 shows how to build a consonant confusion matrix in Tableau, which have already been used in two published studies (Alderete et al., 2019; Alderete, 2022). As with the contingency table, we are interested in counts, so the cells indicate the Number of Records of each confusion. For example, there were 9 speech errors where *p* was confused for *b*. Here we specify the sound field Supplanted Intended as the row dimension, and Sound Intruder as the column dimension, and the filtered values for these fields create the rows and columns. This table is filtered by CV structure (because we only want singleton consonants), master type (restricting the counts to just phonological substitutions), and also the specific values for the intended and intruder sounds (because some of the sounds, like allophonic *ʔ*, are not relevant). Tableau also has certain visualization tools, like scaling the counts with color intensity, to help distinguish frequent from infrequent confusions.

Contingency tables and confusion matrices can be used in a host of ways to explore patterns that relates to key findings in speech error research. For example, the category constraint, the claim that word substitutions tend to be of the same syntactic category (Garrett, 1975), can be explored by creating a confusion matrix on the word fields—Intended Part of Speech and Error Part of Speech—and then analyzed using the diagonal and non-diagonal cells. Likewise, the syllable context constraint (i.e., sounds tend to slip into the same syllabic roles; Boomer and Laver, 1968) can be probed with confusions on the syllabic roles of error and source sounds. The dimensions interface gives direct access to all of the data, as well as tools for shaping searches, and so it is a powerful platform for uncovering speech error patterns.

## 7 Future plans

As a multi-purpose database, SFUSED Cantonese can support a variety of projects, even ones we did not anticipate when creating it. We discuss a few projects that we think can be fruitfully explored below.

Before sketching these projects, it is useful to highlight some of the limitations of the current database to provide context for future plans. First, the conversations the data were drawn from are in the past, so we cannot directly interview the talkers about their intended utterances, as done in some studies (*cf.* Dell, 1984; Harley, 1984). Also, while the example field contains sufficient linguistic material to contextualize the speech error, the current data collection does not include full transcripts of the conversations. Research comparing the speech errors with other speech in the same conversations will therefore require generating transcripts using speech-to-text software. Perhaps the most significant limitation of the database is its size. As a medium-sized database, it has sufficient data to probe the structure of sound errors, but the baselines for word errors and errors of morphological and syntactic processing are much smaller. Research into these areas will likely require a doubling of the current size to 5,000 or 6,000 errors, which the description of methods outlined above makes possible.

Despite these limitations, the unique characteristics of the database support a range of new directions, some of which we sketch here. One aspect of the database that distinguishes it from others is that all of the speech errors come from audio recordings, which opens up new possibilities for speech analysis. Tone and intonation can be probed and examined as a conditioning factor in errors, phonetic facts that have proved useful in error classification (Shattuck-Hufnagel and Cutler, 1999). Disfluencies and other kinds of phonetic cues of uncertainty can also be investigated in connection with error incidence, which has been shown to be important in monitoring studies (Postma, 2000). Finally, recent research has examined the phonetic structure of sound blends in which the properties of the mis-selected target sound are reflected in the error sound (Goldrick and Blumstein, 2006). Cantonese has several consonant series and vowel oppositions that are suitable for a similar kind of probe and can potentially add to the evidence that such phonetic blends occur in spontaneous speech (Alderete et al., 2021) with data from a non-Indo-European language. In sum, access to the underlying acoustic record of the error opens up many new research possibilities.

As alluded to above in section 5, the characterization of the speech planning envelope is somewhat problematic. First, the nature of the context is more complex than assumed in categorical classifications of direction based on anticipations, perseverations, and exchanges. The source sounds that intrude in an error may be both upstream and downstream, requiring a conjunctive value “anticipation + perseveration.” It is also not at all uncommon to find multiple sources both upstream and downstream within realistic planning envelopes, raising the question of whether multiple sources have a combined impact on speech planning. Perhaps the most important problem is that current analysis simply looks for identical units within a specified window. However, this method does not consider the chance occurrence of a source sound in the planning window. For example, Mandarin Chinese has four tones and almost all syllables in Mandarin words are specified for one of these tones. Assuming a 1 in 4 chance probability (ignoring baseline frequency for the moment), the chance rate of one of these tones occurring in the standard six syllable window is extremely high. In other words, using this planning envelope will result in almost all tone errors being contextual. Furthermore, sounds have baseline frequencies, and these are not factored into the analysis of context. For example, *k* is the most frequent consonant in Cantonese, occurring about five times more frequently than *p* in corpora (Li et al., 2023). Surely, the occurrence of *p* in the planning window has lower probability than *k*, and so its occurrence is less likely to be due to chance. But again, this probabilistic information is not included in the analysis of context. We think that SFUSED Cantonese is a good data set to explore these issues because of its rich longform entries and the unlimited ability to investigate context given the existence of audio backup.

Finally, SFUSED Cantonese, as well as SFUSED English, are good data sets for exploring individual differences in language production. SFUSED Cantonese contains speech errors from 21 different talkers, and seven of these have provided 100 or more errors. Using this information, and time metrics created from the audio recordings, one can study individual differences in terms of overall rates and error types. Past research on disordered speech has documented a continuum of speech error patterns that relates to a small number of parameters in the interactive two-step model of language production (Dell et al., 1997; Foygel and Dell, 2000). The existence of audio recordings and the association of specific talkers with each speech error allow us to probe individual differences and determine if they too can be placed on this continuum.

## Data availability statement

The datasets presented in this study can be found in online repositories. The names of the repository/repositories and accession number(s) can be found at: https://osf.io/u58m9.

## Ethics statement

Ethical review and approval were not required for the study on human participants in accordance with the local legislation and institutional requirements. Written informed consent was not required to participate in this study in accordance with the local legislation and institutional requirements.

## Author contributions

JA: Data curation, Methodology, Writing – original draft.

## Appendix

Supporting materials on OSF page (https://osf.io/u58m9/)

The database is available either as a FileMakerPro database (FMP) or raw data (XLSX, CSV):

- sfusedCantoneseVersion_DataSizeDate (FMP, XLSX, CSV): with 2,502 errors or all data

Additional data sets in the sfusedCantonese_data sets folder include:

- dataDataDependencies: tables of systematic relationships among fields
- dataPodcastProspects: list of 36 potential Cantonese podcasts with selection criteria
- dataRecordings: analysis of each recording, including linguistic features by speaker

Materials for training and processing data are available in sfusedCantonese_methods:

- methodsDataAnalysisWorkflow: blueprint of workflow for data analyst
- methodsDataInput: specific documentation procedures for data collectors
- methodsPhoneticTests: Cantonese transcription tests for data collectors
- methodsSubmissionTemplate: spreadsheet template for recording speech errors from audio

Linguistic reference material is available in sfusedCantonese_linguistics:

- linguisticsGrammarSynopsis: Cantonese grammar synopsis
- linguisticsCasualSpeech: documentation of all casual speech rules in Cantonese
- linguisticsSoundCharts: transcription conventions of all Cantonese sounds
